# Risk factors for sepsis: a systematic review and meta-analysis

**DOI:** 10.3389/fpubh.2026.1790141

**Published:** 2026-06-26

**Authors:** Chu Qin, Sisi Lin, Lisha Pang, Mahong Hu, Dieyu Ma, Zhizhen Zhou, Xiujuan Xu, Conghua Ji

**Affiliations:** 1 School of Public Health, Zhejiang Chinese Medical University, Hangzhou, China; 2 Data Center, Sengong Hospital of Shaanxi Province, Xi’an, China; 3 Intensive Care Unit, Tongde Hospital of Zhejiang Province, Hangzhou, China

**Keywords:** incidence, meta-analysis, risk factors, sepsis, systematic review

## Abstract

**Objective:**

This study aims to identify key risk factors for the incidence of sepsis through a systematic review and meta-analysis, thereby providing evidence-based medical evidence for its primary prevention and early identification.

**Methods:**

Systematic searches were conducted across eight Chinese and English databases (CNKI, Wanfang, SinoMed, VIP, PubMed, Web of Science, Embase, Cochrane Library) from their inception to January 2025. Study selection, data extraction, and quality assessment were performed independently by two reviewers. Meta-analysis was then carried out using RevMan software. Effect sizes were pooled using either fixed- or random-effects models based on heterogeneity, and sensitivity analyses were performed for outcomes with high heterogeneity.

**Results:**

A total of 12,536 records were identified, and 23 studies met the eligibility criteria, of which 21 studies were included in the quantitative meta-analysis. The pooled analyses identified 19 significant factors associated with sepsis incidence, including 18 risk factors and 1 protective factor. The risk factors identified were: age (≥60 years and per-year increase), male sex, low BMI (≤18.5), Black race, diabetes, chronic obstructive pulmonary disease (COPD), cancer, kidney disease, postoperative infection, ASA score >2, GCS score <8, ISS (per-point increase), loss of functional independence, steroid use, mechanical ventilation, history of hospitalization, history of emergency surgery, and previous history of sepsis. Antibiotic use was identified as a significant protective factor. Sensitivity analyses revealed that heterogeneity significantly decreased after excluding specific studies for age, diabetes, postoperative infection, and mechanical ventilation.

**Conclusion:**

This study provides a comprehensive review of risk factors for the incidence of sepsis. Correct identification of these risk factors and early implementation of interventions should be prioritized to reduce the incidence of sepsis.

## 1 Introduction

Sepsis is a life-threatening organ dysfunction syndrome caused by a dysregulated host response to infection. It has been identified as a leading cause of preventable mortality in intensive care settings and represents a critical global public health challenge that demands urgent attention (1). The underlying pathological mechanisms of sepsis are highly complex, involving systemic inflammatory response syndrome, coagulation abnormalities, immune dysregulation, tissue damage, and aberrant host responses to pathogenic microorganisms and their toxins. These mechanisms are closely interrelated with multisystem and multi-organ pathophysiological alterations (2–4).

Sepsis represents a substantial global health burden and serves as a leading cause of death among adults in intensive care units (ICUs) (5). Recent global estimates indicate that in 2021 there were about 166 million sepsis cases and 21.4 million sepsis-related deaths worldwide, representing roughly one-third of all global deaths (6). Notably, nearly half of all sepsis cases globally—20 million—occur in children under 5 years of age. Within this age group, the most common causes of sepsis are diarrheal diseases (5.9 million cases), followed by neonatal disorders and lower respiratory infections (7). Although sepsis can develop in any individual with infection, severe trauma, or major non-communicable diseases, the risk is significantly higher among older adults, pregnant women, newborns, hospitalized patients, ICU patients, immunocompromised individuals, and those with chronic medical conditions (8).

Sepsis is characterized by complex risk factors and high post-onset mortality, making the identification of its causative elements crucial for prevention and early clinical intervention. Although numerous studies have investigated potential risk factors for sepsis, existing evidence remains fragmented and heterogeneous, with substantial variations in study populations, clinical settings, and diagnostic criteria. Current meta-analyses on sepsis risk factors have predominantly focused on neonatal populations and are often limited to specific regions, resulting in a notable lack of comprehensive evidence regarding sepsis incidence in the general population (9, 10). Moreover, advances in clinical management and evolving patient demographics and disease profiles further necessitate a re-evaluation of sepsis-related risk factors based on currently available data.

Therefore, this study conducted a meta-analysis to systematically examine factors influencing the occurrence of sepsis, with the aim of establishing a robust evidence-based foundation for the precise identification of high-risk populations and the development of early intervention strategies.

## 2 Methods

### 2.1 Protocol and registration

This study was performed following the Preferred Reporting Items for Systematic Reviews and Meta-Analyses (i.e., “PRISMA”) guidelines (11). It was registered in the International Prospective Register of Systematic Reviews (PROSPERO) under registration number CRD420251124533. As a secondary analysis, this study did not require ethical approval.

### 2.2 Database and search strategies

We selected a combination of Chinese and English databases to ensure comprehensive coverage of both national and international literature. Chinese databases (CNKI, Wanfang, SinoMed, and VIP) were included to capture studies published in Chinese journals, which are often not indexed in international databases. English databases (PubMed, Web of Science, Embase, and the Cochrane Library) were chosen to identify studies published in international peer-reviewed journals covering clinical medicine, epidemiology, and evidence-based research. All databases were searched from their inception to January 2025. The search strategy for PubMed is provided as an example:

#1 “Sepsis” [Mesh] OR “Shock, Septic” [Mesh].

#2 “Risk” [Mesh] OR “Risk Assessment” [Mesh] OR “predict*” [Title/Abstract].

#3 “Case–Control Studies” [Mesh] OR “Cohort Studies” [Mesh].

#4 “Morbidity” [Mesh] OR “Incidence” [Mesh].

#5 #1 AND #2 AND #3 AND #4.

The search strategy combined both controlled vocabulary (MeSH terms) and free-text keywords to maximize sensitivity. Key headings such as “Sepsis” and “Shock, Septic” captured all relevant disease terms, while “Risk,” “Risk Assessment,” and “predict*” targeted studies evaluating risk factors. Study design terms (“Case–Control Studies,” “Cohort Studies”) were used to restrict inclusion to analytical studies suitable for meta-analysis, and outcome terms (“Morbidity,” “Incidence”) ensured that the retrieved studies reported the occurrence of sepsis as the primary outcome.

### 2.3 Inclusion criteria

(1) The study population consisted of general adult participants aged ≥ 18 years;
(2) Participants were free from sepsis at enrollment;
(3) The study outcome was the occurrence of sepsis, including severe sepsis and septic shock;
(4) The study provided results from multivariate regression analysis on risk factors for the incidence of sepsis, including odds ratios (OR), hazard ratios (HR), or risk ratios (RR);
(5) Study designs were limited to case–control or cohort studies.

### 2.4 Exclusion criteria

(1) Case reports, review articles, and animal studies;
(2) Studies for which the full text could not be retrieved;
(3) Studies with incomplete data that precluded quantitative meta-analysis;
(4) Duplicate publications.

### 2.5 Screening of literature

All retrieved records were exported and managed using EndNote X9.3.3 reference management software for screening purposes. Duplicate citations were first removed automatically by the software, followed by a manual review by the investigators to identify and exclude any remaining duplicates not detected by the automated process. After deduplication, an initial screening was conducted based on titles and abstracts to exclude references that were clearly irrelevant to the study objectives (including mismatches in population or study design). The remaining articles underwent full-text review against the predefined inclusion and exclusion criteria. The entire screening and selection process was carried out independently by two investigators. Any discrepancies between reviewers were resolved through discussion to reach consensus. If agreement could not be achieved, a third researcher was consulted to make a final decision.

Data extraction was performed using Microsoft Excel 2021. The following key information was collected: first author, publication year, country, patient type, patient source, study design, sample size, number of sepsis cases, sex ratio, effect measures, and primary outcome indicators. Two investigators independently extracted the data, which was subsequently cross-checked and verified for accuracy. In case of disagreement, it was first resolved through discussion between the two reviewers. If consensus could not be reached, a third researcher was consulted to make the final judgment.

### 2.6 Risk of bias

The methodological quality and risk of bias of the included studies were assessed using the Newcastle-Ottawa Scale (NOS) (12). The NOS evaluates observational studies across three key domains: the selection of study groups, the comparability of groups, and the ascertainment of exposure or outcome. These domains are further subdivided into a total of nine items. The total possible score on the NOS is 9 points. Based on their scores, studies were classified into three quality categories: low quality (scores 0–3), indicating a high risk of bias; moderate quality (scores 4–6), indicating a moderate risk of bias; and high quality (scores 7–9), indicating a low risk of bias. Two investigators independently performed the quality assessments, with any disagreements resolved through consultation with a third reviewer.

### 2.7 Statistical methods

All statistical analyses were performed using RevMan 5.4.1. Pooled OR or RR were calculated for predictors of sepsis incidence along with heterogeneity testing. When the event rate was low (< 20%), RR values were considered approximate to OR. Statistical heterogeneity among studies was assessed using the I^2^ statistic. In accordance with the recommendations by Tufanaru et al. (13), a fixed-effect model was applied when five or fewer studies were available for a given outcome. For analyses involving more than five studies, the choice between fixed-effect or random-effects models was determined based on the I^2^ value: a random-effects model was used if I^2^ > 50%, indicating substantial heterogeneity; otherwise, a fixed-effect model was employed.

### 2.8 Sensitivity analysis

If significant heterogeneity is observed among the study results, sensitivity analysis will be employed to explore the sources of heterogeneity.

### 2.9 Publication bias

Given that the number of studies included for each outcome in this analysis did not exceed 10, quantitative assessment of publication bias was not performed.

## 3 Results

### 3.1 Literature screening

**Figure 1:**
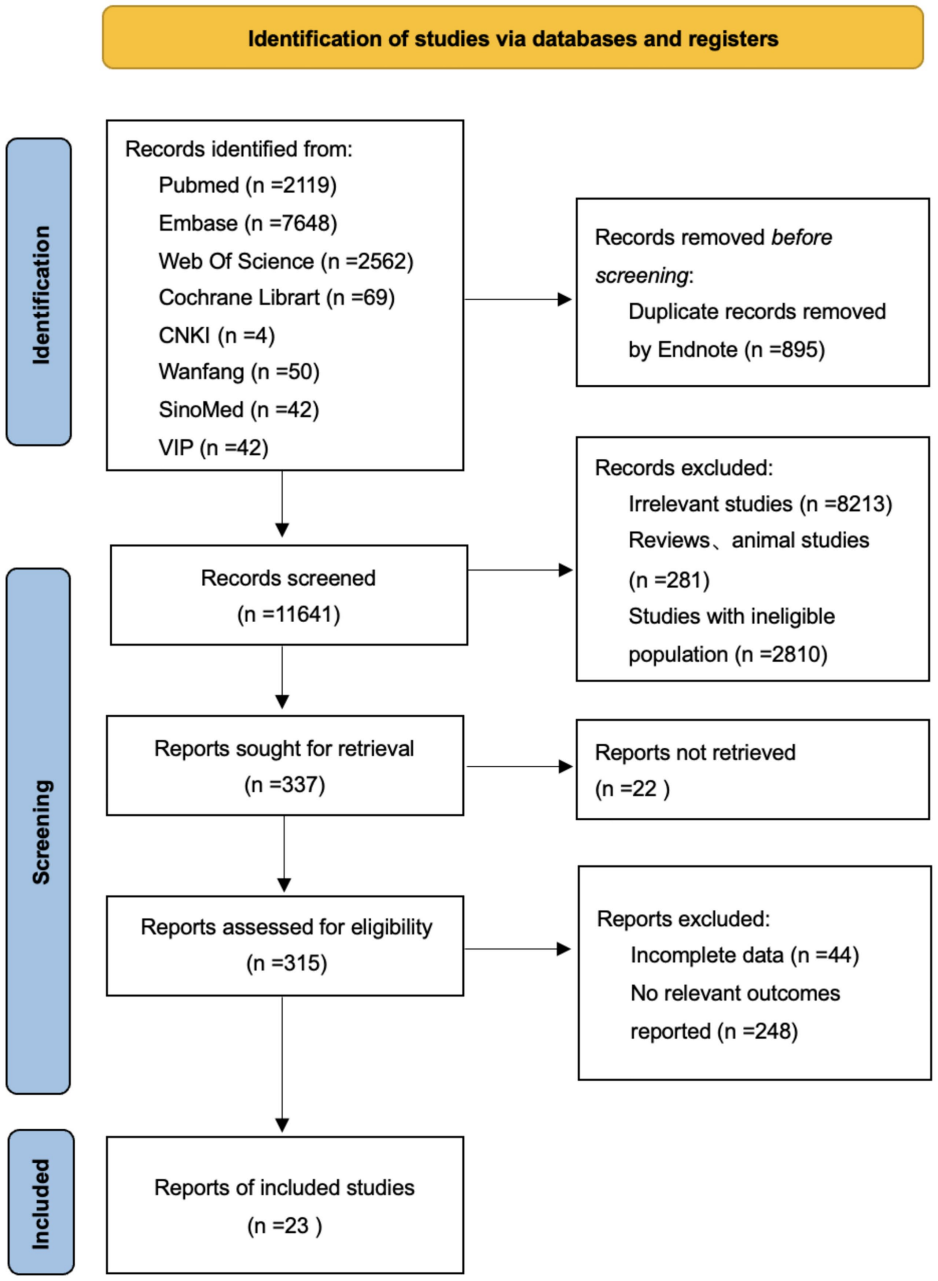
PRISMA flow diagram of study selection.

A total of 12,536 records were identified through systematic searches across eight databases. After removing 895 duplicates using EndNote software, 11,641 unique records remained. Following initial screening based on titles and abstracts, 11,304 articles were excluded for the following reasons: 8,213 were irrelevant to the research focus, 281 were animal studies or reviews, and 2,810 involved ineligible study populations. Full-text retrieval and assessment were conducted for the remaining 337 articles. Among these, 22 were excluded due to unavailability of full text, 248 lacked outcomes of interest, and 44 had incomplete data. Ultimately, 23 studies met the eligibility criteria and were included in this analysis. The 23 included studies were published between 2011 and 2024. Study numbers gradually increased from 1 to 2 per year in the early years to 2–4 per year in 2015–2017, and have remained relatively stable (1–3 per year) since 2019. Overall, while there is a slight upward trend in the volume of research over time, there is no strong surge in the number of publications in the most recent years. The study selection process is detailed in Figure 1.

### 3.2 Study characteristics

Table 1 summarizes the characteristics of the included studies investigating risk factors for the incidence of sepsis. Among the 23 studies identified (14–36), 15 utilized a retrospective cohort design and 8 were prospective cohort studies. The study populations encompassed surgical patients (11 studies, 47.83%), individuals with infections (4 studies, 17.39%), patients with advanced chronic diseases (3 studies, 13.04%), cancer patients (2 studies, 8.69%), and other groups including those with chronic obstructive pulmonary disease (1 study, 4.35%), dementia (1 study, 4.35%), and amyotrophic lateral sclerosis (1 study, 4.35%). Geographically, the majority of studies were conducted in the United States (8 studies, 34.78%), followed by China (5 studies, 21.74%), Germany (2 studies, 8.69%), Spain (2 studies, 8.69%), and single studies from Pakistan, Brazil, South Korea, Canada, Thailand, and Iran (each 1 study, 4.35%). The diversity in study populations and geographic regions likely reflects the inherently heterogeneous and globally prevalent nature of sepsis. As a syndrome resulting from a dysregulated host response to infection, sepsis can arise in diverse clinical settings, including surgical care, chronic illnesses, malignancies, and acute infections (37). This broad clinical spectrum contributes to its worldwide occurrence and explains why sepsis has been extensively studied across different countries and healthcare systems. Including studies from varied clinical backgrounds and geographic regions enhances the generalizability of our findings and provides a more comprehensive understanding of sepsis risk factors across diverse patient populations.

**Table 1:** Characteristics and extracted data from included studies.

First author	Year	Country	Patient characteristics	Setting	Study design	Sample size, n	Sepsis definition	Sepsis cases, n	Gender (male/female)	Effect measure	Risk factors
Li, Qiang (14)	2024	China	Liver cirrhosis	Inpatient	Retrospective cohort study	136	Sepsis-3	35	81/55	OR	1, 2, 3, 4, 5, 6, 7, 8, 9, 10
Waqar, U.	2024	Pakistan	Thyroidectomy	Inpatient, outpatient	Retrospective cohort study	180,373	Sepsis-1	247	37,018/143,355	OR	2, 11, 12, 13, 14, 15, 16, 17, 18, 19, 20, 21, 22, 23
Borno, H. T.	2023	United States	COVID-19 with invasive cancer	Inpatient, outpatient, ICU	Prospective cohort study	303	-	30	147/156	OR	3, 11, 24
Gonzalez, Christian A.	2023	United States	Hip fracture surgery	Inpatient	Retrospective cohort study	86,438	Sepsis-1	1,361	26,882/59,556	OR	2, 11, 13, 14, 25, 26, 27, 28, 29
Bhojani, Naeem	2022	Canada	Ureteroscopic surgery	Inpatient, ICU	Retrospective cohort study	104,100	ICD-9, ICD-10	5,706	50,271/53,829	OR	1, 2, 6, 11, 12, 19, 30, 31, 32
Chan, Yi-Chia	2022	China	End-stage liver disease requiring transplantation	Inpatient	Retrospective cohort study	342	Sepsis-1	36	259/83	OR	5, 33, 34
Pertsch, N. J.	2020	United States	Neurosurgery	Inpatient	Retrospective cohort study	122,466	Sepsis-1	1,067	57,689/64,777	OR	1, 3, 11, 12, 13, 20, 14, 25, 26, 35
Ninh, A.	2019	United States	Appendectomy	Inpatient	Prospective cohort study	72,538	Sepsis-1	311	36,237/36,301	OR	1, 3, 6, 11, 12, 2, 1, 36, 37,
Yeh, Liang-Tsai	2019	China	Dementia	Inpatient	Retrospective cohort study	4,980	ICD-9	51	226/276	HR	11
Zaid, Y.	2019	Iran	Severe ischemic stroke	ICU	Prospective cohort study	122	Sepsis-1	76	52/70	OR	25, 38, 39, 40, 41, 42
Chaudhary, Ninad S.	2017	United States	Hospital-acquired infection	Inpatient	Retrospective cohort study	131	Sepsis-1	96	-	OR	26
Lakomkin, N.	2017	United States	Orthopedic trauma	Inpatient	Prospective cohort study	361,402	Sepsis-1	2,524	162,819/198,583	OR	11, 13, 14, 26, 32, 43, 44, 45
Lee, Cynthia Wei-Sheng (26)	2017	China	Amyotrophic lateral sclerosis	Inpatient	Retrospective cohort study	3,505	ICD-9	294	2,155/1,350	HR	1, 19, 43, 46, 47
Ruiz-Mesa, J. D.	2017	Spain	Acute pyelonephritis or complicated acute pyelonephritis	Emergency department	Prospective cohort study	1,507	Sepsis-1	423	793/714	OR	1, 48, 49, 50, 51, 52, 53
Montull, B.	2016	Spain	Community-acquired pneumonia	Inpatient	Prospective cohort study	4,070	Sepsis-1	1,529	3,240/830	OR	1, 2, 5, 25, 52, 54
Park, J. H.	2016	South Korea	Trauma	ICU	Retrospective cohort study	182	Sepsis-1	15	127/55	OR	1, 6, 55
Khamnuan, P.	2015	Thailand	Necrotizing fasciitis	Emergency department	Retrospective cohort study	1,452	Sepsis-1	237	818/633	OR	1, 2, 11, 56, 57, 58, 59
Liao, K. M.	2015	China	COPD	Inpatient, ICU	Retrospective cohort study	6,740	ICD-9	805	4,443/2,297	OR	1, 19, 37, 43, 52, 60, 61,
MacQueen, I. T.	2015	United States	Abdominal and pelvic surgery	Inpatient	Retrospective cohort study	478	Sepsis-1	33	-	OR	3, 6, 20, 21
Sammon, J. D.	2015	United States	Major cancer surgery	Inpatient	Retrospective cohort study	2,502,710	ICD-9	47,551	15,091,134/993,576	OR	1, 6, 11, 36, 41, 47, 62
Berger, B.	2014	Germany	Ischemic or hemorrhagic stroke	ICU	Retrospective cohort study	238	Sepsis-1	30	163/75	OR	7, 25, 63
Elias, A. C.	2012	Brazil	Surgical patients	ICU	Prospective cohort study	625	Sepsis-1	158	352/273	OR	6, 20, 43, 64, 65
Wafaisade, A.	2011	Germany	Polytrauma	ICU	Prospective cohort study	29,829	Sepsis-1	3,042	22,020/7,809	OR	1, 6, 11, 24, 38, 55, 66, 67, 68

Primary outcomes: 1. Age; 2. Diabetes; 3. Cancer; 4. Liver disease; 5. Antibiotic use; 6. Surgical history; 7. Acute Physiology and Chronic Health Evaluation II (APACHE II) score; 8. Alkane level; 9. Saturated fat; 10. Lipid metabolism value; 11. Sex; 12. BMI; 13. ASA score; 14. Loss of functional independence; 15. Smoking; 16. Pulmonary disease; 17. Surgical incision type; 18. Surgical indication; 19. History of hospitalization; 20. Operation time; 21. Postoperative infection; 22. Pneumonia; 23. Urinary tract infection; 24. Number of comorbidities; 25. COPD; 26. Steroid use; 27. Anemia; 28. Leukocytosis; 29. Hypoalbuminemia; 30. Hyperlipidemia; 31. Elixhauser Comorbidity Index; 32. Previous sepsis; 33. Child-Pugh classification; 34. Hepatic hydrothorax; 35. Bleeding disorder; 36. Ethnicity; 37. Acute renal failure or dialysis; 38. GCS score; 39. Acute Physiology and Chronic Health Evaluation IV (APACHE IV) score; 40. National Institutes of Health Stroke Scale (NIHSS) score; 41. Length of hospital stay; 42. Acute Physiology Score (APS); 43. Mechanical ventilation; 44. Preoperative resting pain; 45. Hypertension; 46. Amyotrophic lateral sclerosis (ALS); 47. Charlson Comorbidity Index (CCI); 48. Previous episode of complicated acute pyelonephritis; 49. Previous urinary tract instrumentation; 50. Dysuria syndrome; 51. Urinary tract dilation; 52. Kidney disease; 53. Bacteremia; 54. Alcohol abuse; 55. ISS; 56. Heart disease; 57. Herpes infection; 58. Skin necrosis; 59. Total protein; 60. Dementia; 61. Vasopressor use; 62. Health insurance; 63. Immunosuppressive disease; 64. Fluid resuscitation; 66. Vasoactive drugs; 66. Number of surgical procedures; 67. Units of red blood cells transfused; 68. Injury severity.

In this study, all types of sepsis were included without restriction to specific subtypes or etiologies. Detailed information on the definition of sepsis used in each study is provided in Table 1.

The included studies reported a total of 68 factors potentially associated with the incidence of sepsis. However, due to insufficient data availability—such as variables reported in only a single study (e.g., amyotrophic lateral sclerosis, anemia, Child-Pugh classification), inconsistent cutoff values or units across studies, reversed control group definitions, or heterogeneous effect measures—meta-analysis was not feasible for the majority of these factors. Consequently, only 19 factors from 21 studies (14–21, 23–25, 27–36) were included in the quantitative synthesis. Most of these studies were retrospective cohort designs and provided adjusted effect estimates. These studies primarily contributed data on key clinical characteristics and comorbidities, forming the main evidence base for the pooled analyses. Detailed contributions of individual studies are presented in Table 1. These factors encompassed patient age, sex, body mass index (BMI), ethnicity, diabetes, malignancy, chronic obstructive pulmonary disease (COPD), renal disease, postoperative infection, functional independence, American Society of Anesthesiologists (ASA) score, Glasgow Coma Scale (GCS) score, Injury Severity Score (ISS), steroid use, antibiotic use, history of hospitalization, history of emergency surgery, previous sepsis, and mechanical ventilation.

### 3.3 Risk of bias

The methodological quality of the 21 studies included in the meta-analysis was assessed using the NOS. The overall NOS scores ranged from 5 to 9, with 11 studies (52.38%) scoring 7 or higher, indicating generally moderate to high methodological quality. Most studies performed well in the selection and comparability domains. In contrast, points were most frequently deducted in the outcome domain, especially regarding the adequacy and duration of follow-up. Notably, only one study achieved the maximum score of 9, indicating that although the overall quality was acceptable, limitations related to follow-up assessment and reporting were common. These limitations may contribute to residual bias and partially explain the heterogeneity observed in the pooled analyses. Detailed scoring is provided in Table 2.

**Table 2:** Quality assessment of included studies.

First author	Year	Selection				Comparability		Outcome			Total quality
		Representativeness of the exposed cohort	Selection of the non-exposed cohort	Ascertainment of exposures	Demonstration that outcome of interest was not present at start of study	The study controlled for the most important factor?	The study controlled for any additional factor?	Assessment of outcome	Adequacy of follow up of cohort	Adequacy of follow up of cohort	
Li, Qiang	2024	1	1	0	0	1	1	1	0	0	5
Waqar, U.	2024	1	1	1	0	1	1	1	0	0	6
Borno, H. T.	2023	1	1	0	0	1	1	1	0	0	5
Gonzalez, Christian A.	2023	1	1	1	1	1	1	1	0	0	7
Chan, Yi-Chia	2022	1	1	1	0	1	1	1	1	1	8
Bhojani, Naeem	2022	1	1	1	0	1	1	1	0	0	6
Pertsch, N. J.	2020	1	1	1	0	1	1	1	0	0	6
Zaid, Y.	2019	1	1	1	1	1	1	1	0	0	7
Ninh, A.	2019	1	1	1	1	1	1	1	0	0	7
Chaudhary, Ninad S.	2017	1	1	1	0	1	0	1	1	1	7
Ruiz-Mesa, J. D.	2017	1	1	1	0	1	1	1	0	1	7
Lakomkin, N.	2017	1	1	1	1	1	1	1	0	0	7
Park, J. H.	2016	1	1	1	0	1	1	1	0	0	6
Montull, B.	2016	1	1	1	0	1	1	1	1	0	7
Khamnuan, P.	2015	1	1	1	1	1	1	1	1	1	9
Sammon, J. D.	2015	1	1	1	0	1	1	1	0	0	6
MacQueen, I. T.	2015	1	1	1	0	1	1	1	0	0	6
Liao, K. M.	2015	1	1	1	0	1	1	1	0	0	6
Berger, B.	2014	1	1	1	0	1	1	1	0	0	6
Elias, A. C.	2012	1	1	1	1	1	1	1	0	0	7
Wafaisade, A.	2011	1	1	0	1	1	1	1	0	0	6

### 3.4 Results of the meta-analysis

#### 3.4.1 Age

**Figure 2:**
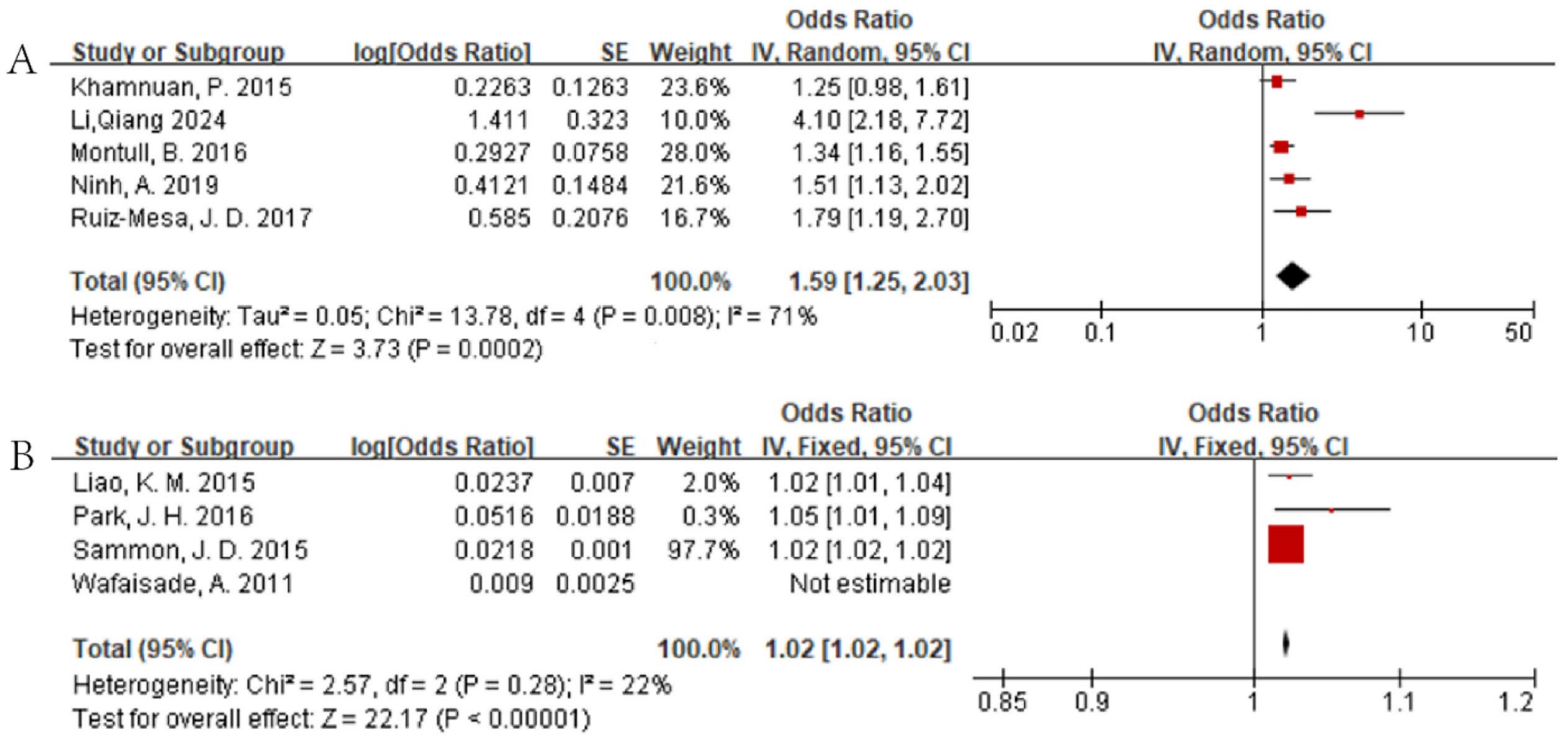
Effect of age on sepsis onset. **(A)** Association between age ≥ 60 years and sepsis onset; **(B)** association between each additional year of age and sepsis onset.

Nine studies investigated the impact of age on the incidence of sepsis. Pooled analysis revealed a significantly higher risk of sepsis among patients aged ≥ 60 years compared to their younger counterparts, as shown in Figure 2. Age of 60 years or older was identified as a risk factor for sepsis (OR: 1.59; 95% CI: 1.25–2.03; I^2^ = 71%). Notably, considerable heterogeneity was observed across the included studies (I^2^ = 71%), which may reflect variations in population characteristics or clinical settings among the studies.

**Figure 3:**
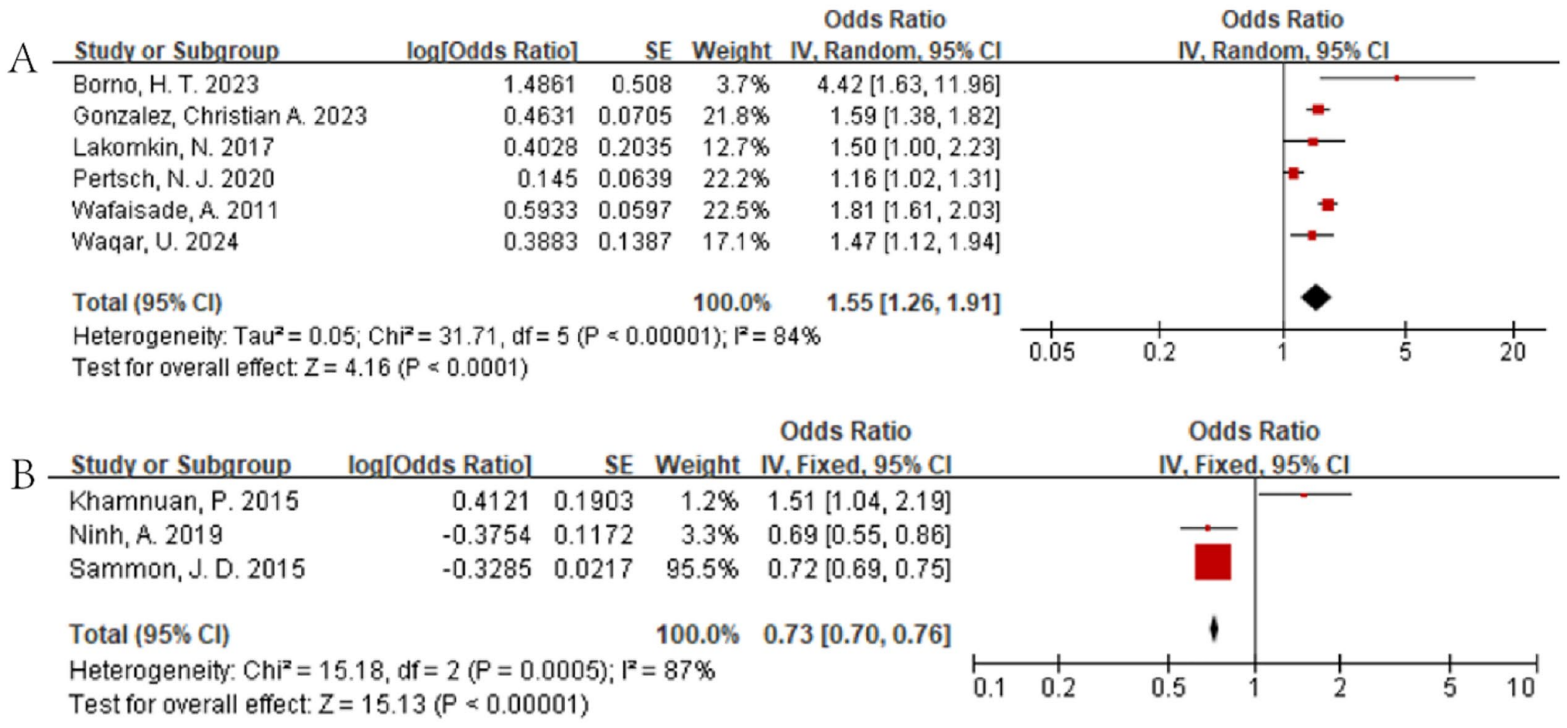
Effect of sex on sepsis onset. **(A)** Male sex; **(B)** female sex.

Figure 3 further refined this association. Specifically, each additional year of age was associated with a 2% increase in sepsis risk, indicating a positive correlation between advancing age and sepsis incidence (OR: 1.02; 95% CI: 1.02–1.02; I^2^ = 22%), with low heterogeneity observed This precise year-by-year escalation underscores that aging represents not only a categorical threshold (≥60 years) but also a continuous physiological process of accumulating risk.

#### 3.4.2 Sex

Nine studies examined the association between sex and sepsis onset. Of these, six studies reported on the effect of male sex, while three studies reported on the effect of female sex. The pooled analysis identified male sex as a risk factor for sepsis (OR: 1.55; 95% CI: 1.26–1.91; I^2^ = 84%). Conversely, female sex was found to be a protective factor (OR: 0.73; 95% CI: 0.70–0.76; I^2^ = 87%), as shown in Figure 3.

#### 3.4.3 Comorbidities

**Figure 4:**
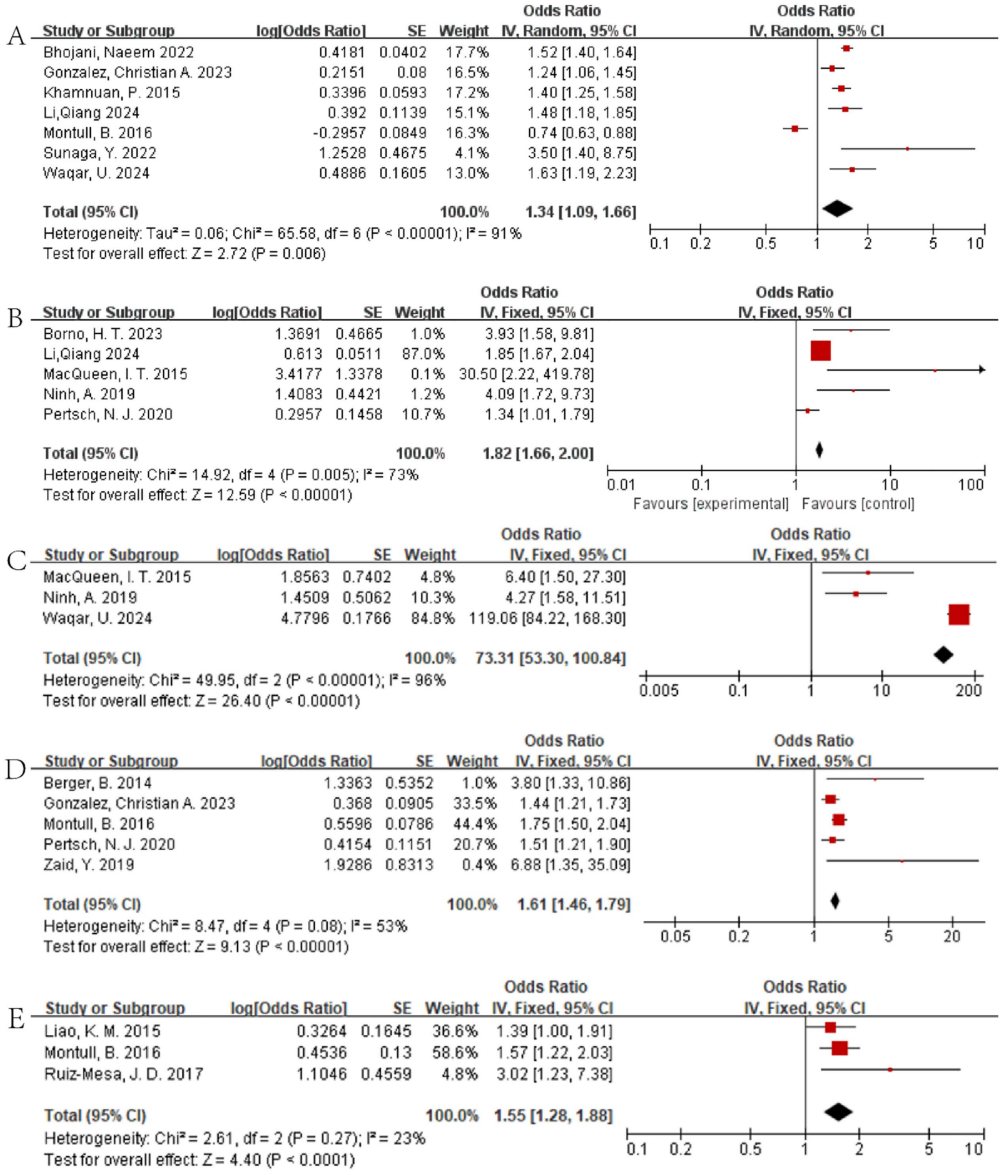
Effect of comorbidities on sepsis onset. **(A)** Diabetes; **(B)** cancer; **(C)** postoperative infection; **(D)** COPD; **(E)** kidney disease.

Five key comorbidities were evaluated for their association with sepsis onset. The pooled analyses demonstrated that all examined conditions were significant risk factors for sepsis, with varying effect sizes and precision, as summarized in Figure 4.

Specifically, postoperative infection was associated with the highest risk of sepsis (OR: 73.31; 95% CI: 53.3–100.84, I^2^ = 96%). Among chronic conditions, cancer showed the strongest association (OR: 1.82; 95% CI: 1.66–2.00; I^2^ = 73%), followed by COPD (OR: 1.61; 95% CI: 1.46–1.79; I^2^ = 53%), diabetes (OR: 1.34; 95% CI: 1.09–1.66; I^2^ = 91%), and kidney disease (OR: 1.55; 95% CI: 1.28–1.88, I^2^ = 23%).

#### 3.4.4 Clinical scores

**Figure 5:**
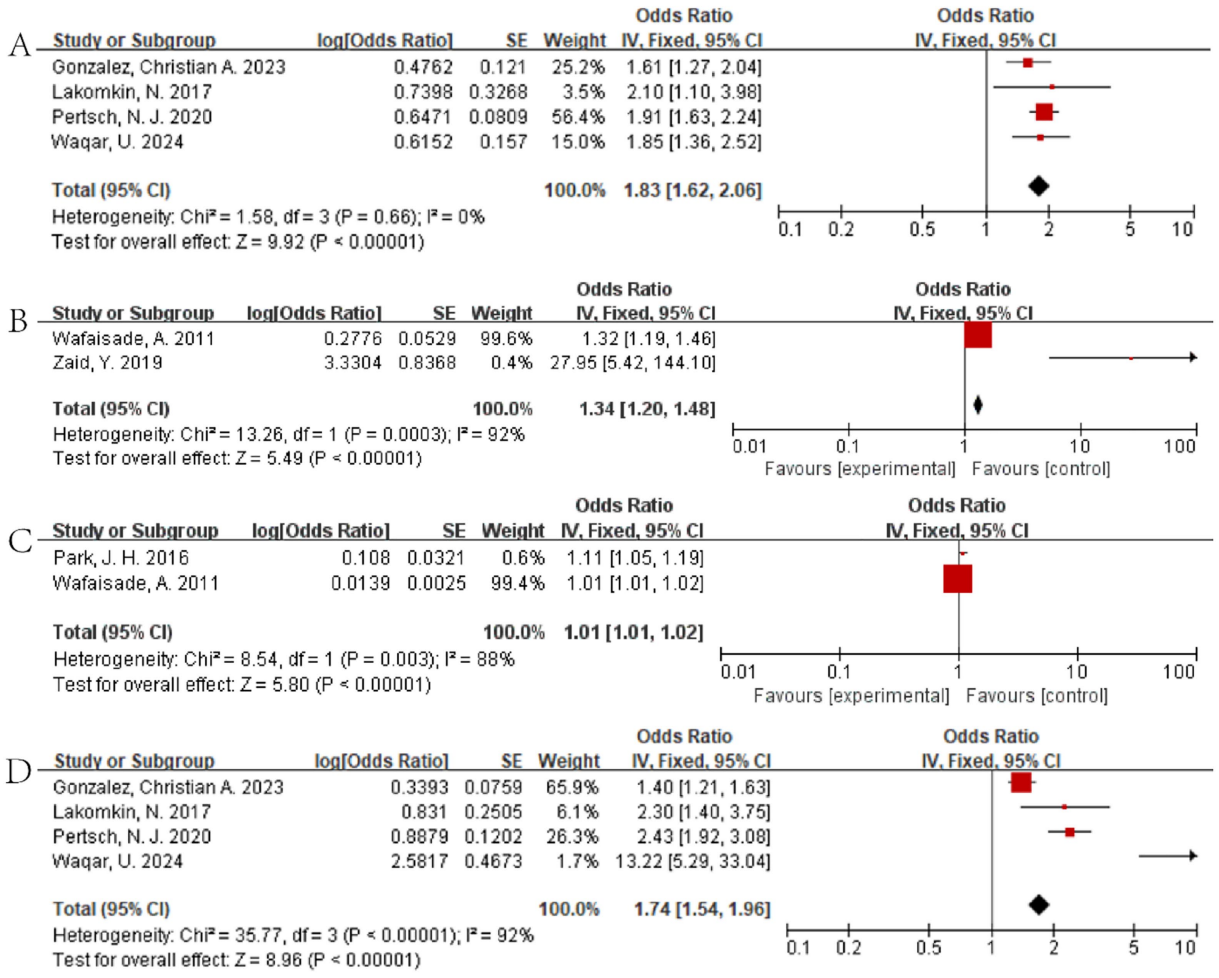
Effect of clinical scores on sepsis onset. **(A)** ASA score; **(B)** GCS score; **(C)** ISS; **(D)** Functional independence.

The impact of four clinical assessment scores on sepsis risk was evaluated. The pooled analyses demonstrated that higher severity or impairment across all scoring systems was consistently associated with an increased risk of sepsis, as summarized in Figure 5.

Specifically, an ASA score > 2 was associated with a significantly higher risk of sepsis (OR: 1.83; 95% CI: 1.62–2.06, I2 = 0%). Similarly, a GCS score < 8 (OR: 1.34; 95% CI: 1.20–1.48) and loss of functional independence (OR: 1.74; 95% CI: 1.54–1.96, I^2^ = 92%) were identified as significant risk factors. Furthermore, each unit increase in the ISS was associated with a slight but significant increase in sepsis risk (OR: 1.01; 95% CI: 1.01–1.02).

#### 3.4.5 Treatments

**Figure 6:**
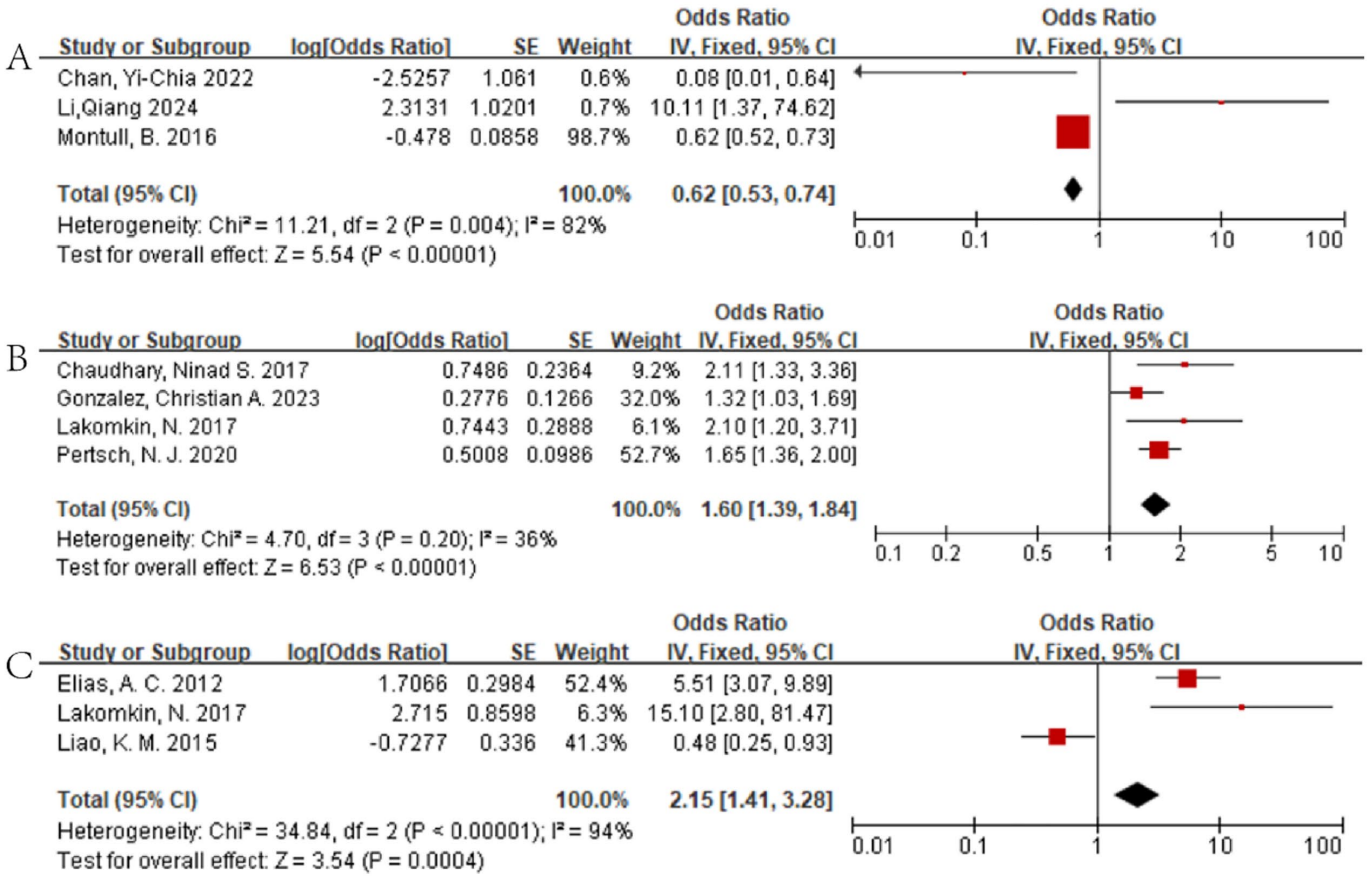
Effect of treatments on sepsis onset. **(A)** Antibiotic use; **(B)** steroid use; **(C)** mechanical ventilation.

Three key treatment measures were evaluated for their association with sepsis onset. The pooled analyses yielded distinct patterns: one intervention was protective, while two were associated with significantly increased risk, as summarized in Figure 6.

Antibiotic use was identified as a significant protective factor against sepsis (OR: 0.62; 95% CI: 0.53–0.74; I^2^ = 82%). In contrast, both steroid use (OR: 1.60; 95% CI: 1.39–1.84; I^2^ = 36%) and mechanical ventilation (OR: 2.15; 95% CI: 1.41–3.28; I^2^ = 94%) were associated with a significantly higher risk of developing sepsis, with mechanical ventilation demonstrating the strongest effect size among all treatment factors examined.

#### 3.4.6 Other factors

**Figure 7:**
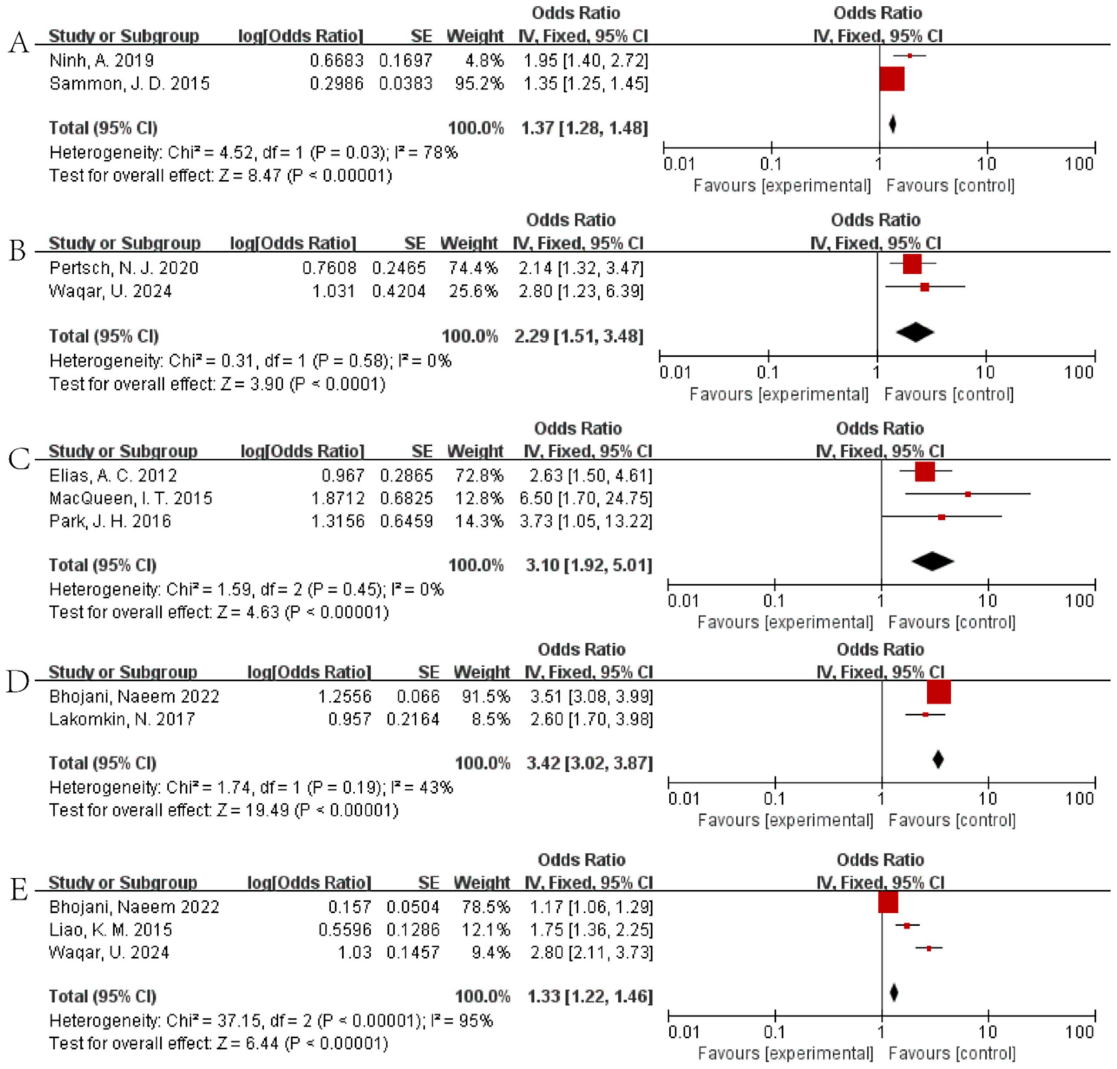
Effect of other factors on sepsis onset. **(A)** Black race; **(B)** BMI; **(C)** History of emergency surgery; **(D)** History of sepsis; **(E)** History of hospitalization.

Five additional factors were evaluated for their association with sepsis onset. The pooled analyses demonstrated that all were significant risk factors, with a notably strong effect observed for a history of prior sepsis, as summarized in Figure 7.

The strongest predictor was a history of sepsis, which was associated with more than a threefold increase in risk (OR: 3.42; 95% CI: 3.02–3.87; I^2^ = 43%). Similarly, a history of emergency surgery was associated with a substantially elevated risk (OR: 3.10; 95% CI: 1.92–5.01; I^2^ = 0%). Low body mass index (BMI ≤ 18.5) was also a strong risk factor (OR: 2.29; 95% CI: 1.51–3.48; I^2^ = 0%). More modest, yet statistically significant, increases in risk were associated with Black race (OR: 1.37; 95% CI: 1.28–1.48; I^2^ = 78%) and a history of hospitalization (OR: 1.35; 95% CI: 1.24–1.47; I^2^ = 93%).

### 3.5 Sensitivity analysis

For pooled results with substantial heterogeneity, sensitivity analyses were performed by sequentially excluding individual studies to determine whether the overall findings were influenced by any single study and to explore potential sources of heterogeneity. High heterogeneity was observed for the following factors: age, sex, diabetes, cancer, postoperative infection, functional independence, antibiotic use, mechanical ventilation, and history of hospitalization. Sensitivity analysis was conducted for each of these factors. The results revealed that for age, diabetes, postoperative infection, and mechanical ventilation, the heterogeneity significantly decreased after the removal of a particular study, suggesting that study may be a source of heterogeneity. After excluding the studies contributing to heterogeneity, the pooled ORs and 95% CIs were as follows: 1.38 (1.23–1.54) for age, 1.45 (1.31–1.59) for diabetes, 4.86 (2.14–11.01) for postoperative infection, and 6.14 (3.53–10.67) for mechanical ventilation. The recalculated OR values after exclusion were adopted as the final pooled results in the meta-analysis. Table 3 summarizes the factors for which heterogeneity significantly decreased following sensitivity analysis. For the remaining factors, heterogeneity did not decrease with the sequential exclusion of studies, indicating robust and stable results.

**Table 3:** Sensitivity analysis of risk factors for the incidence of sepsis.

Risk factor	Study excluded	OR	95% CI	I^2^
Age		1.59	1.25–2.03	71.00%
	Khamnuan, P. 2015	1.76	1.27–2.45	76.00%
	Li, Qiang 2024	1.38	1.23–1.54	0.00%*
	Montull, B. 2016	1.77	1.22–2.55	76.00%
	Ninh, A. 2019	1.66	1.20–2.29	78.00%
	Ruiz-Mesa, J. D. 2017	1.56	1.18–2.07	76.00%
Diabetes		1.34	1.09–1.66	91.00%
	Bhojani, Naeem 2022	1.32	1.01–1.73	90.00%
	Gonzalez, Christian A. 2023	1.37	1.07–1.77	92.00%
	Khamnuan, P. 2015	1.35	1.02–1.79	92.00%
	Li, Qiang 2024	1.32	1.04–1.69	92.00%
	Montull, B. 2016	1.45	1.31–1.59	48.00%*
	Sunaga, Y. 2022	1.29	1.04–1.59	92.00%
	Waqar, U. 2024	1.34	1.09–1.66	91.00%
Postoperative infection		73.31	53.30–100.84	96.00%
	MacQueen, I. T. 2015	82.96	59.84–115.03	97.00%
	Ninh, A. 2019	101.71	72.64–142.43	93.00%
	Waqar, U. 2024	4.86	2.14–11.01	0.00%*
Mechanical ventilation		2.15	1.41–3.28	94.00%
	Elias, A. C. 2012	0.76	0.41–1.41	93.00%
	Lakomkin, N. 2017	1.88	1.22–2.92	97.00%
	Liao, K. M. 2015	6.14	3.53–10.67	19.00%*

*Removal of this study resulted in a significant reduction in the overall heterogeneity of the Risk factor.

### 3.6 Summary of results

Table 4 summarizes the influencing factors and their effect magnitudes on the incidence of sepsis.

**Table 4:** Risk factors for the incidence of sepsis.

Risk factor	OR	95% CI
Age (≥ 60 years)	1.38	1.23–1.54
Age (per year)	1.02	1.02–1.02
Sex (male)	1.55	1.26–1.91
Sex (female)	0.73	0.70–0.76
BMI (≤ 18.5)	2.29	1.51–3.48
Black race	1.37	1.28–1.48
Diabetes	1.45	1.31–1.59
COPD	1.61	1.46–1.79
Cancer	1.82	1.66–2.00
Kidney disease	1.55	1.28–1.88
Postoperative infection	4.86	2.14–11.01
ASA score (> 2)	1.83	1.62–2.06
GCS score (< 8)	1.34	1.20–1.48
ISS (per-point)	1.01	1.01–1.02
Functional independence	1.74	1.54–1.96
Steroid use	1.60	1.39–1.84
Antibiotic use	0.62	0.53–0.74
Mechanical ventilation	6.14	3.53–10.67
History of hospitalization	1.35	1.24–1.47
History of emergency surgery	3.10	1.92–5.01
Previous history of sepsis	3.42	3.02–3.87

## 4 Discussion

This study employed systematic review and meta-analysis to integrate relevant risk estimates and odds ratios, providing the highest level of evidence-based synthesis on influencing factors for sepsis incidence. The findings offer evidence for the primary prevention and early identification of sepsis.

Although numerous studies worldwide have investigated risk factors for sepsis, key issues remain, including substantial heterogeneity in reported factors and lack of uniform cutoff criteria. This study systematically screened 68 factors associated with the incidence of sepsis. However, due to significant variations in measurement methods and cutoff values across studies for certain factors, as well as an insufficient number of studies for others, quantitative meta-analysis was only feasible for 19 predictive factors associated with sepsis incidence.

From the results, the following 19 factors were identified as significant predictors of sepsis in hospitalized patients: age, sex, BMI, race, diabetes, COPD, cancer, kidney disease, postoperative infection, ASA score, GCS score, ISS, functional independence, steroid use, antibiotic use, mechanical ventilation, history of hospitalization, history of emergency surgery, and previous history of sepsis.

Among these, age—the most frequently reported risk factor—was strongly associated with the incidence of sepsis in hospitalized patients. Advanced age, particularly in older adult patients, is accompanied by declined immune function, increased comorbidities, and reduced physiological reserve, all of which significantly elevate not only the risk of developing sepsis but also the likelihood of poor outcomes. Studies suggest that immunosenescence and a chronic inflammatory state in the older adults are major contributors to their heightened susceptibility to sepsis (38), while these factors also markedly increase sepsis-related mortality. One study demonstrated that mortality among sepsis patients rises with age, with the most pronounced increase observed in individuals aged 71 to 77 years (39). Beyond the independent effect of age, chronic comorbidities commonly present in older adults—such as diabetes, cardiovascular disease, and chronic lung disease—as well as frailty, not only increase vulnerability to infection (40) but also impair the ability to cope with sepsis, leading to worse prognoses (41, 42).

A sensitivity analysis was conducted to explore the heterogeneity surrounding age as a risk factor for sepsis. The results showed that after excluding the study by Li (14), the heterogeneity of the pooled results decreased to 0%. Further analysis indicated that the older age of the population in that study, which used 70 years as a cutoff, may have been a source of heterogeneity. Nevertheless, the findings of the present study remain consistent with those of Li et al., supporting that age is an independent risk factor for sepsis.

Numerous studies consistently indicate that males have a higher susceptibility to sepsis than females. For instance, one study conducted in Sub-Saharan Africa and another from India both demonstrated a significantly greater risk of sepsis among males (9, 10). Furthermore, evidence also suggests that male survivors of sepsis face a higher risk of rehospitalization compared to female survivors (43). These epidemiological observations may be partly explained by underlying biological differences in immune regulation. Estrogen enhances both innate and adaptive immune responses and modulates inflammatory signaling, whereas androgens may exert relatively immunosuppressive effects, potentially resulting in less efficient pathogen clearance in men (44). However, a large prospective cohort study based on the UK Biobank (45) reported that although the overall incidence of sepsis hospitalization was higher in men, several risk factors, such as chronic obstructive pulmonary disease, dyslipidemia, myocardial infarction, and smoking, conferred disproportionately greater relative risk in women. These findings indicate that under certain comorbidity profiles, women may experience amplified vulnerability to sepsis, underscoring the importance of accounting for sex-specific interactions in risk stratification models.

This study demonstrated that postoperative infection, diabetes, cancer, and kidney disease are significant predictors of sepsis incidence. Postoperative infection, a major sepsis risk factor, drives TNF-*α*-mediated inflammation. Rice bran peptides (KF-8) counteract this by enriching *Akkermansia muciniphila* and suppressing TNF-α. This suggests potential gut microbiota modulation via KF-8 as a dual approach to reduce sepsis risk and protect cognitive function postoperatively (46). In patients with diabetes, chronic hyperglycemia can lead to alterations in vascular diameter, resulting in hypoperfusion of vital organs. Moreover, elevated blood glucose levels may exacerbate inflammatory responses and impair immune cell function, thereby increasing the risk of infection and promoting the development of sepsis (47).

Sepsis and cancer share numerous pathophysiological features, as both conditions arise from the host’s immune system failing to adequately respond to the initial insult—whether pathogen invasion in the case of sepsis, or malignant cell transformation in cancer (48). Cancer patients are at significantly increased risk of infection due to immunosuppression caused by the disease itself and its treatments, such as chemotherapy and radiotherapy. Although anticancer therapies have markedly improved survival rates, they come at the cost of elevated risk of life-threatening infectious complications. Therapy-induced acquired immunosuppression is a major contributor to the heightened susceptibility to infections in cancer patients, and infections—including sepsis—have become a leading cause of mortality in this population (49). Studies have shown that the risk of sepsis increases nearly tenfold across all cancer types (50). This elevated risk is influenced by multiple factors, including cancer type, disease stage, and treatment modality. For instance, patients with hematologic malignancies and advanced-stage cancer exhibit particularly high incidence rates of sepsis (51). A recent cohort study of 4,858 sepsis patients with malignancy further refines this risk profile. The study confirms that beyond cancer type, specific clinical factors at admission, including a higher Clinical Frailty Scale score, elevated Sequential Organ Failure Assessment score, and the presence of pulmonary infection, are independent predictors of 30-day mortality (52). Furthermore, cancer patients often experience malnutrition and frailty, which further compromise the host’s defense against infections and increase the likelihood of sepsis development (53).

Furthermore, kidney disease is a significant comorbidity contributing to both the incidence of sepsis. Studies indicate that patients with kidney disease often exhibit chronic inflammation and immune dysfunction (54), which predispose them to infections and facilitate the progression to sepsis. Notably, the relationship between sepsis and renal injury is bidirectional and complex. Sepsis is frequently accompanied by insulin resistance, and an elevated triglyceride-glucose index, serving as a reliable surrogate marker thereof, is an independent risk factor for acute kidney injury in septic patients (55). This implies that metabolic assessment should be integrated into the management of septic patients with kidney disease risk, as metabolic dysregulation may fuel renal injury and sustain a sepsis–kidney injury cycle.

From a public health perspective, diabetes, cancer, and chronic kidney disease—as modifiable risk factors for both the incidence and mortality of sepsis—present critical opportunities for reducing sepsis occurrence. Although these chronic conditions involve distinct pathological mechanisms, they share the common features of being monitorable and manageable. Implementing multi-level prevention and control strategies can effectively mitigate the risk of sepsis.

For instance, in the case of diabetes, an integrated management system spanning the entire course of the disease should be established. At the community level, health education should be enhanced in accordance with the Standards of Medical Care in Diabetes (56), promoting nutritional therapies such as healthy dietary patterns (e.g., the Mediterranean diet) and the development of individualized meal plans, along with encouraging regular physical activity. Research indicates that an energy-reduced Mediterranean diet combined with physical activity can improve bone mineral density in older adults with metabolic syndrome, thereby preserving musculoskeletal health and potentially reducing overall frailty (57). Overmore, this approach targets a key mechanism in metabolic diseases: chronic low-grade inflammation (58). As noted in the Fifth International Consultation on Sexual Medicine (ICSM 2024) (59), diabetes and related conditions drive inflammation that damages vascular endothelium, reducing genital blood flow and contributing to sexual dysfunction such as erectile disorder and low libido. Thus, lifestyle interventions not only improve metabolic and musculoskeletal health but also help preserve vascular and sexual function, supporting overall wellbeing. Primary healthcare providers should be trained to identify high-risk patients, and digital tools should be utilized to establish early warning systems. Furthermore, efforts should be intensified to promote diabetes screening and emphasize glycemic control and the management of microvascular complications (60).

ASA score, GCS score, and ISS are significant predictors of sepsis. Higher ASA scores suggest greater physiological compromise and could be associated with increased sepsis risk, possibly due to reduced functional reserve and higher comorbid burden (61). Lower GCS scores often reflect impaired consciousness and neurological dysfunction, which may contribute to aspiration risk, inadequate airway protection, and altered immune responses, potentially elevating susceptibility to sepsis (62). Previous meta-analyses have similarly reported significantly lower GCS values among trauma patients who developed sepsis, corroborating our finding that GCS < 8 is a clinically meaningful threshold for identifying high-risk individuals (63). Higher ISS values indicate more severe multisystem trauma and tissue damage, which might promote a pro-inflammatory state that could facilitate infectious complications and sepsis development (64). These scoring systems provide objective, standardized metrics that enhance sepsis risk stratification, facilitate early clinical intervention. Although the effect size per ISS point appears modest, its cumulative impact in severely injured patients is clinically relevant (63).

Among the other risk factors, a history of sepsis is the strongest risk factor. Dragoescu et al. (65) help explain this by showing that sepsis induces a persistent state of systemic inflammation and immunothrombosis, reflected in biomarkers like the C-reactive protein-to-platelet ratio, which integrates inflammation and coagulation dysfunction. This suggests sepsis survivors may have chronic low-grade inflammation and endothelial disruption, reducing physiological reserve and amplifying subsequent risk. Emergency surgery carries similar risk. This is because surgery triggers major inflammation and coagulation. In post-sepsis patients, this trauma exacerbates their pre-existing endothelial vulnerability and immune dysregulation, impairing the controlled healing process emphasized by Yan et al. (66). The result is a synergistic increase in risk.

From a methodological perspective, the sensitivity analysis in this study represents a development over earlier research approaches. In the study by de Groot et al. (67), sensitivity analysis was primarily employed to test the robustness of main outcomes by constructing predefined subgroup models—a verification strategy aimed at known confounding variables. In contrast, this study adopts a systematic “leave-one-out” analysis to identify sources of heterogeneity. By sequentially excluding individual studies and observing changes in effect sizes and heterogeneity indices, we were able to pinpoint specific studies contributing to high heterogeneity. This transforms sensitivity analysis from a robustness check into a diagnostic tool, refining pooled estimates and clarifying outcome discrepancies.

However, this study has several limitations. First, the meta-analysis was limited to factors frequently reported in the original studies, and some outcome measures were excluded from pooled analysis due to an insufficient number of available studies, which may affect the comprehensiveness and reliability of the findings. Second, to ensure the accuracy of the pooled results, only studies reporting effect estimates using ORs were included for the predictive factors of sepsis incidence. This selection approach may have introduced a certain degree of bias. Although numerous studies reported factors associated with sepsis prognosis and infection, incomplete or insufficiently detailed data reporting prevented their inclusion in the meta-analysis, leading to a potential loss of information.

Future research should prioritize large-scale, well-designed prospective cohort studies to validate the risk factors for sepsis identified in this meta-analysis, using standardized sepsis definitions and consistent measurement strategies to reduce heterogeneity. Leveraging electronic health records and real-world data, advanced artificial intelligence and machine learning approaches can further refine risk stratification. For instance, hybrid federated learning combined with explainable AI has been shown to optimize sepsis mortality prediction while preserving data privacy and model interpretability (68). In addition, machine learning models integrating correlated community-level sociodemographic and socioeconomic variables have demonstrated value in clarifying population-level determinants of sepsis incidence (69). Integrating these computational strategies with precision medicine frameworks, such as individualized risk profiling and dynamic prediction models, may enhance early identification and enable more targeted prevention across both community and hospital settings.

## 5 Conclusion

This systematic review and meta-analysis identified 19 significant predictors of sepsis incidence, including 18 risk factors and one protective factor. Age, comorbidities such as diabetes, cancer, COPD, and kidney disease, as well as clinical severity indicators including higher ASA score, lower GCS score, and higher ISS, were consistently associated with increased sepsis risk. In addition, treatment-related factors—such as postoperative infection, mechanical ventilation, steroid use, and emergency surgery—further contributed to sepsis development. These findings highlight that sepsis risk is multifactorial, involving demographic characteristics, baseline health status, disease severity, and healthcare-related exposures. Early identification of high-risk individuals based on these predictors may facilitate targeted monitoring and timely preventive interventions, ultimately improving clinical outcomes.
